# Detection of *Pseudomonas aeruginosa* Metabolite Pyocyanin in Water and Saliva by Employing the SERS Technique

**DOI:** 10.3390/s17081704

**Published:** 2017-07-25

**Authors:** Olga Žukovskaja, Izabella Jolan Jahn, Karina Weber, Dana Cialla-May, Jürgen Popp

**Affiliations:** 1 Institute of Physical Chemistry and Abbe Center of Photonics, Friedrich Schiller University Jena, Helmholtzweg 4, 07743 Jena, Germany; olga.zukovskaja@leibniz-ipht.de (O.Ž.); karina.weber@leibniz-ipht.de (K.W.); 2 Research Campus InfectoGnostics, Philosophenweg 7, 07743 Jena, Germany; 3 Leibniz Institute of Photonic Technology Jena, Albert-Einstein-Str. 9, 07745 Jena, Germany; izabella.jahn@leibniz-ipht.de

**Keywords:** SERS, pyocyanin, microfluidics, diagnosis, artificial sputum

## Abstract

Pyocyanin (PYO) is a metabolite specific for *Pseudomonas aeruginosa*. In the case of immunocompromised patients, it is currently considered a biomarker for life-threating *Pseudomonas* infections. In the frame of this study it is shown, that PYO can be detected in aqueous solution by employing surface-enhanced Raman spectroscopy (SERS) combined with a microfluidic platform. The achieved limit of detection is 0.5 μM. This is ~2 orders of magnitude below the concentration of PYO found in clinical samples. Furthermore, as proof of principle, the SERS detection of PYO in the saliva of three volunteers was also investigated. This body fluid can be collected in a non-invasive manner and is highly chemically complex, making the detection of the target molecule challenging. Nevertheless, PYO was successfully detected in two saliva samples down to 10 μM and in one sample at a concentration of 25 μM. This indicates that the molecules present in saliva do not inhibit the efficient adsorption of PYO on the surface of the employed SERS active substrates.

## 1. Introduction

Increasing antibiotic resistance impedes the successful treatment of infections, such as pneumonia or tuberculosis. This leads to longer hospital stays, higher medical costs and increased mortality [1]. Therefore, it is essential to identify the exact pathogen prior to the antibiotic treatment.

*Pseudomonas aeruginosa* is an important gram-negative bacterium and is the leading cause of respiratory tract infections in the case of patients with compromised host defense mechanisms. Therefore, this pathogen is responsible for the highest rates of acquired infections in intensive-care units [2]. Because of its intrinsic ability to develop antibiotic resistance, to form impenetrable biofilms and to release a large arsenal of virulence factors [3], *P. aeruginosa* is one of the greatest therapeutic challenges, and rapid detection, as well as the selection of the appropriate antibiotic to initiate the therapy, is critical to optimize the clinical outcome.

Currently, the routine detection and identification of *P. aeruginosa* in respiratory samples is performed by bacteriological culture. The time to achieve reliable results is at least 24 h. An alternative approach is the indirect detection of the bacteria by identifying the biomarkers released by the organism. *P. aeruginosa* produces several toxic metabolites, the most predominant of them being the redox-active phenazine compound pyocyanin (PYO). PYO is capable of destroying host cells by altering critical cellular processes, generating reactive oxygen species and inducing neutrophil apoptosis [4]. *P. aeruginosa* is the only microorganism known to produce PYO, and for this reason PYO can be used as a potential biomarker. It was found that in clinical sputum samples PYO can reach concentrations up to 16.5 μg/mL (~76 μM) for cystic fibrosis patients and up to 27.3 μg/mL (~130 μM) for patients with bronchiectasis [5]. Moreover, Hunter et al. concluded that levels of PYO and its biosynthetic precursor, phenazine-1-carboxylic acid, varying between 7.7 μM and 46.8 μM in expectorated cystic fibrosis sputum samples are negatively correlated with lung function [6]. The presence and the concentration of PYO in clinical samples is traditionally determined through extraction of the pigment with chloroform and by subsequently performing high performance liquid chromatography (HPLC). However, HPLC is available only in academic and clinical centers and it is time consuming, costly and laborious.

Surface-enhanced Raman spectroscopy (SERS) was proven to be a molecular specific and highly sensitive analytical technique suitable for biological applications [7,8,9,10]. SERS allows the sensitive identification of the molecular fingerprint of the analyte brought in the proximity of plasmonic nanostructures by enhancing its Raman signal by several orders of magnitude. For example, Polisetti et al. demonstrated that SERS can be used for investigating PYO produced by laboratory and cystic fibrosis lung isolate strains of *P. aeruginosa* for different growing conditions [11]. Additionally, Bodelon et al. developed different Au nanorod structures and performed SERS measurements for studying the intercellular communication of growing *P. aeruginosa* biofilms on the basis of secreted PYO [12]. Further on, the detection of PYO in clinical sputum samples was also successfully performed using silver nanorod array substrates [13]. However, due to the complex sputum matrix, PYO was outcompeted for available adsorption sites on the metallic surface by other species and its SERS detection became possible only after extracting it from sputum samples by the traditional chloroform procedure. Additionally, even though SERS is a highly specific and sensitive detection method, traditional SERS measurements still suffer from low reproducibility. This hinders a quantitative analysis. Combining SERS with microfluidics overcomes this limitation and enables the handling of small sample volumes [14,15,16].

Within this study, SERS was first combined with a droplet-based microfluidic platform to obtain reproducible and automated measurement conditions for the detection of PYO in aqueous environment. This is essential because, as the majority of the already published SERS studies have demonstrated, for each targeted analyte, a smart experimental design is needed to obtain SERS spectra with an optimized signal-to-noise ratio [17,18,19]. By taking into account the ability of the biomarker to induce the aggregation of the employed silver nanoparticles and by carefully choosing the measurement design, PYO levels down to concentrations of 0.5 μM were detected with the lab-on-a-chip SERS (LoC-SERS) platform. In the second part of the study, saliva was considered to be a clinically relevant sample matrix. This body fluid can be collected in a simple, non-invasive manner and offers high chemical complexity. Additionally, when collecting sputum, the clinical samples also contain saliva. This makes saliva an important and easy-to-access sample. Consequently, the interferences brought by this matrix in the SERS spectra have to be assessed. The results demonstrate that despite the high complexity of the matrix, PYO spiked in saliva collected from healthy volunteers can be successfully detected in the micromolar range.

## 2. Materials and Methods

### 2.1. Chemicals and Reagents

Pyocyanin from *P. aeruginosa*, ≥98% (HPLC) in powder form, silver nitrate (ACS reagent, 99%), hydroxylamine hydrochloride (ReagentPlus, 99%) and mineral oil were purchased from Sigma Aldrich (St. Louis, MO, USA), whereas potassium chloride (KCl) (≥99.5% p.a. ACS, ISO) was acquired from Carl Roth (Karlsruhe, Germany).

### 2.2. Silver Nanoparticles (Ag NPs) Synthesis

The silver colloidal solution was prepared through the chemical reduction of silver nitrate by hydroxylamine in the presence of sodium hydroxide at room temperature, as established in the standard Leopold and Lendl protocol [20]. The synthesized nanoparticles were characterized by UV-Vis spectroscopy (Figure S1) and transmission electron microscopy (TEM) (Figure S2).

### 2.3. Pyocyanin Solution

Since PYO is sparingly soluble in water, first a stock solution of PYO at 1 mM concentration was prepared in ethanol by adding the appropriate amount of powder. Working solutions were obtained by diluting the stock solution with high-purity water, keeping a concentration of 10% of ethanol in the final solutions.

### 2.4. Saliva Sample Preparation

Saliva collected from the control group of the IDES (Innovative Diagnostik für Infectionserregern bei Pneumonien, InfectoGnostics research campus (13GW0096F)) study (ethical approval 4672-01/16) were used. The samples were obtained from two female and one male volunteer at least 1 h after a meal. The samples were stored at 4 °C until analysis. None of the individuals had undergone medical treatment at the time of sample collection and they were also not in medical evidence for chronic diseases. Prior to the measurements, PYO solutions in ethanol with different concentrations were added to the saliva samples in a ratio of 1 to 9. The as obtained samples had the following biomarker concentration: 10, 25, 50, 75 and 100 μM. As negative control, a sample containing only ethanol and saliva was considered. To remove oral epithelial cells and food debris, the samples were filtrated using a cellulose acelate membrane with a pore size of 0.2 μm (VWR International, Radnor, PA, USA).

### 2.5. Instrumentation

Raman and SERS spectra were acquired with a commercially available WITec Raman microscope (WITec GmbH, Ulm, Germany) equipped with a diode laser (Fandango, Cobolt) that emits at 514 nm. During the measurements, a 600 lines per mm grating was used with a spectral resolution of ~5 cm^−1^ and the same objective (Nikon 20 × 0.4 N.A., Tokyo, Japan) was employed for focusing the laser beam on the sample and for collecting the Raman backscattered light. For all measurements, a laser power of 38 mW and an integration time of 1 s were used. The reference Raman and SERS spectra of the water-ethanol, as well as of the PYO solution were measured in plastic cuvettes and each spectrum is the result of 50 accumulations.

**Scheme 1:**
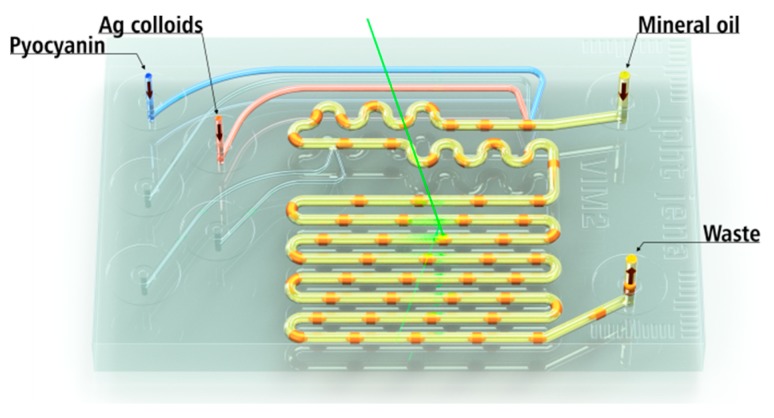
Schematic representation of the droplet based microfluidic chip used for LoC-SERS measurements.

In order to determine the limit of detection of PYO in aqueous solution, a droplet-based glass microfluidic chip was employed (Scheme 1).

A detailed description of the chip has been published elsewhere [21]. The glass syringes (ILS Germany GmbH, Stützerbach, Germany) were filled with different solutions and connected with the chip via Teflon capillaries, having an inner diameter of 0.5 mm (WICOM GmbH, Heppenheim, Germany). The solutions were pumped into the chip via a computer controlled pump system (neMESYS Cetoni GmbH, Korbußen, Germany). To create a segmented continuous flow, mineral oil was pumped with a flow rate of 9 nL/s, while the analyte and the Ag NPs were both injected with 7 nL/s. All other ports were blocked. The SERS spectra were measured continuously with an integration time of 1 s.

To assess the interferences brought by the molecules present in the saliva, the control samples and the ones spiked with PYO were mixed with the silver colloidal solution in a 1 to 1 ratio, followed by the addition of 1 M KCl (final mixing ratio 1:1:0.2). Here, KCl was added to induce the aggregation process. Three independent measurements were recorded for each sample with 1 s integration time and 20 accumulations.

### 2.6. Data Processing

The data processing was performed using an algorithm developed in-house in the programming language R [22]. In the case of the LoC-SERS measurements, Raman/SERS spectra were measured continuously during the flow. Accordingly, alternating SERS spectra of the analyte containing droplets, Raman spectra of the oil phase and mixed spectra of both phases were recorded. Therefore, during the data processing, the oil and mixed spectra were removed first. Afterwards, spectra were background corrected using the Statistics-sensitive Non-linear Iterative Peak-clipping (SNIP) algorithm with 80 iterations and cut to the region of interest [23]. Univariate statistical analysis was performed by calculating the peak area of the band around 676 cm^−1^. This Raman mode corresponds to the ring breathing mode of PYO. For peak integration, the Simpson’s rule [24] was applied. The integrated peak area was plotted as function of the concentration. SERS spectra measured in plastic cuvettes were vector-normalized. The same background correction algorithm was also applied to the spectra recorded in cuvettes.

## 3. Results and Discussion

### 3.1. SERS Detection of Pyocyanin in Aqueous Solution

**Scheme 2:**
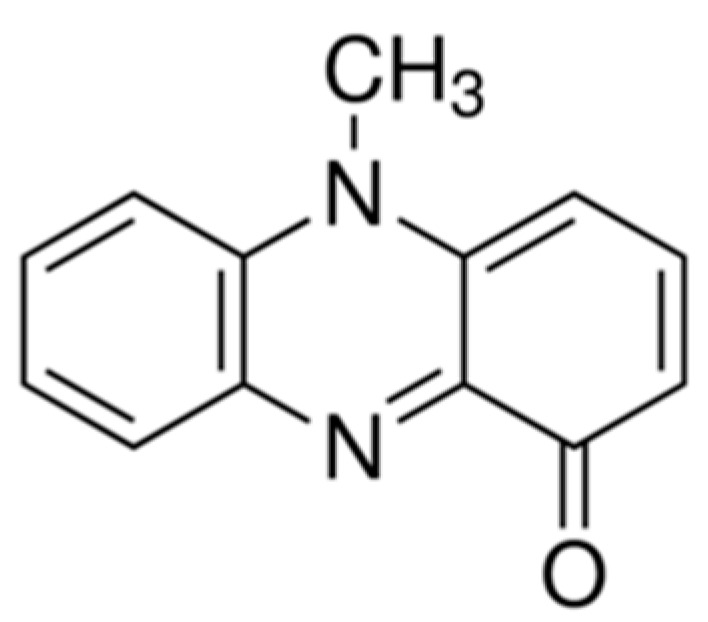
Chemical structure of the PYO molecule (C_13_H_10_N_2_O).

PYO is a redox-active phenazine and its chemical structure is depicted in Scheme 2. It is a heterocyclic nitrogen containing compound, which exist as a zwitterion at the pH value of blood (7.35–7.45). PYO can accept a single electron, yielding a relatively stable anion radical, and readily undergoes a redox cycle [25].

**Figure 1:**
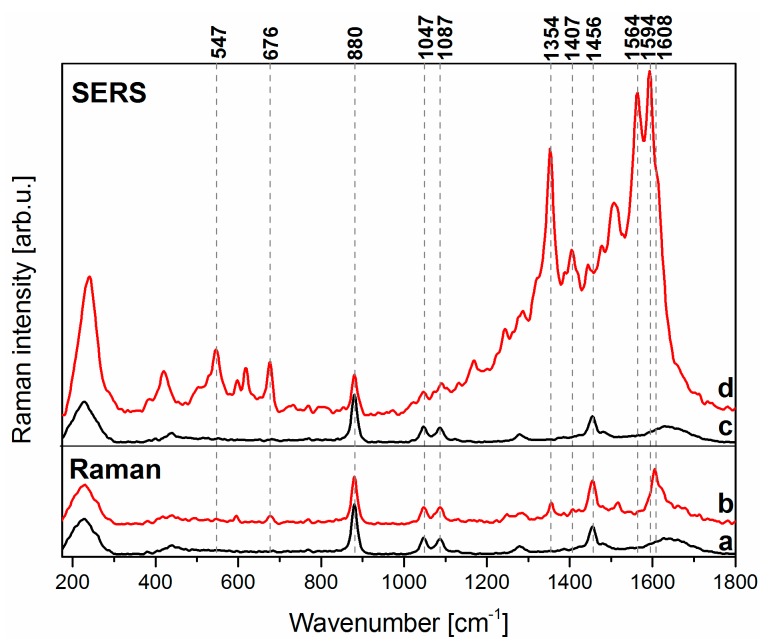
Normalized Raman spectra of 10% ethanol solution (a) and PYO 500 μM solution (b), as well as normalized SERS spectra of the same ethanol solution (c) and PYO 10 μM solution (d).

In Figure 1, the normalized Raman spectrum of PYO in a water-ethanol solution at a concentration of 0.5 mM and the normalized SERS spectrum of PYO at a concentration of 10 μM are plotted together with the Raman and the SERS spectra of the solvent without the analyte. Based on this, the normal Raman spectrum of the PYO solution is dominated by the Raman modes assigned to the molecular vibrations of ethanol at 880, 1047, 1087 and 1456 cm^−1^ (see Figure 1a). Nevertheless, at 676, 1353 and 1606 cm^−1^ clearly distinguishable Raman bands ascribed to the presence of the PYO molecule can be also noticed. A comprehensive band assignment of the Raman modes of the metabolite can be found in the literature [12,13]. Briefly, the band around 676 and 1606 cm^−1^ was assigned to the ring deformations, while the signal at 1354 cm^−1^ corresponds to the combined C-C stretching, C-N stretching and C-H in-plane bending modes of the aromatic ring. In the presence of the Ag nanoparticles, the already mentioned Raman marker bands are strongly enhanced and new ones appear. More exactly, the double band at 1564 cm^−1^ and 1594 cm^−1^, ascribed to the ring deformation and the C-C stretch vibration, the two bands at 423 and 547 cm^−1^, corresponding to the ring torsion and the ring breathing with C-N torsion, become prominent, whereas the Raman band at 1606 cm^−1^ is convoluted with the rest of the signal.

### 3.2. LoC-SERS Measurement of PYO in Aqueous Solution

In order to achieve automated measurements and to obtain reproducible SERS spectra of PYO in aqueous solution at different concentrations, the microfluidic setup depicted in Scheme 1 was employed. Generally, to get strong Raman signal enhancements, the aggregation of spherical nanoparticles is required [26]. Usually the creation of “hot-spots” is achieved by the addition of an active (e.g., KCl) or a passive (e.g., KNO_3_) electrolyte to the colloid-analyte mixture. However, based on our performed experiments, PYO induces by itself the aggregation of the colloids and the addition of aggregation agents is not needed. This is demonstrated by recording the SERS spectra in the absence of salts as well as in the presence of KCl at different concentrations (Figure S3). For clarity, the integrated peak area of the Raman band located at ~676 cm^−1^ is represented in the inset of Figure S3 for different measurement conditions. This band was chosen because its intensity is not influenced by surrounding bands. According to the results, the most intensive and stable signal is achieved when no salts are present. The decrease of the signal with KCl addition can be explained by the fact that the presence of KCl causes over-aggregation and creates large clusters, which cannot support high electromagnetic enhancements at the used excitation wavelength. Therefore, all following measurements were carried out in the absence of any additional aggregation agent.

Another important factor for SERS measurements is the time elapsed between the induction of the aggregation and the measurements. Performing measurements in the microfluidic chip offers time-control by choosing the measurement position. For this purpose, during SERS measurements, the excitation laser was focused in the middle of each microfluidic channel. The results showed that the intensity of the signal was the same for different positions, but the lowest relative standard deviation of the signal was achieved for the measurement done in the 3rd channel (Figure S4). Therefore, all subsequent measurements were performed with the laser beam fixed in the middle of the 3rd channel.

**Figure 2:**
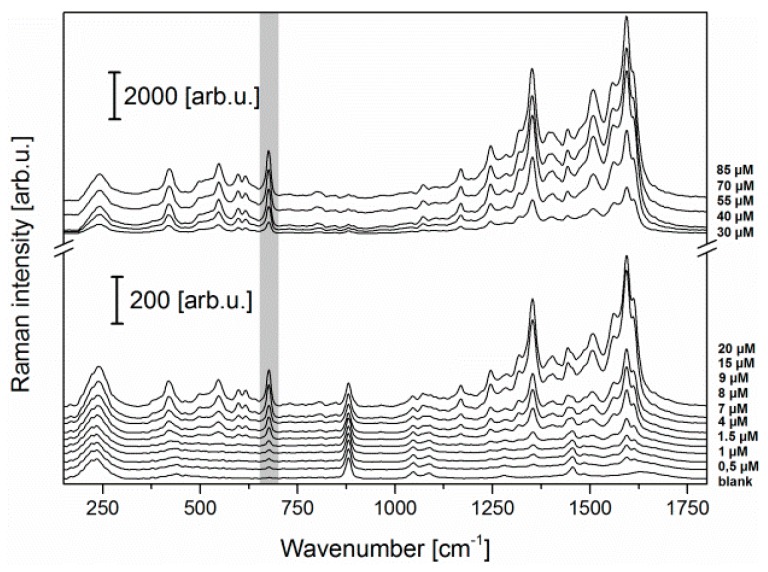
Mean SERS spectra of PYO with concentrations between 0.5 and 85 μM in aqueous solution measured in the microfluidic platform. The mean spectrum of the blank (when only water–ethanol solution is pumped through at the first dosing unit) is also presented.

**Figure 3:**
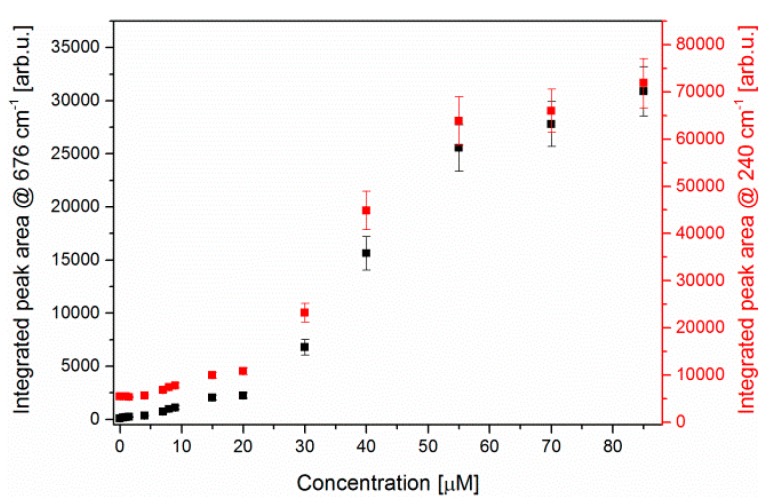
Integrated peak area of the PYO Raman mode at 676 cm^−1^ (black) and of the mode at 240 cm^−1^ which corresponds to the Ag-(adsorbed atom) (red).

By taking the clinically relevant range into account (7–130 μM) [5,6], PYO solutions with concentrations between 0.5 μM and 85 μM were measured employing our proposed LoC-SERS-based detection scheme. In Figure 2, the mean SERS spectra for all concentration steps are plotted together with the blank spectrum. It can be observed that for concentrations higher than 20 μM, the most prominent ethanol band at 880 cm^−1^ becomes convoluted with the intense PYO spectra. The spectral signature of PYO at 676 cm^−1^ and 1353 cm^−1^ is still present at a concentration of 0.5 μM. For better visualization, the integrated peak area of the ring deformation vibration band at 676 cm^−1^ was plotted against the concentration of PYO (Figure 3). The concentration curve has a sigmoidal form, reaching a plateau for concentrations higher than 55 μM. This is explained by a full occupation of the Ag nanoparticle’s surface with PYO molecules. Thus, by further increasing the concentration of the PYO molecules, no significant increase of the band intensity was observed due to an increased distance of the analyte molecule toward the metallic surface.

It is known that usually the bonding of molecules to the silver surface takes place either through the lone pair electrons of the nitrogen or oxygen atoms or through the π electron system of the aromatic ring [27,28]. In the first two cases, Raman bands assigned to the vibrational modes of the Ag-adsorbed molecule (i.e., Ag-N, Ag-O) can appear in the low wavenumber region (200–260 cm^−1^) because of the molecule adsorption. Furthermore, when electrolytes containing Cl^−^ are used for inducing the aggregation of the nanoparticles, a low wavenumber Raman band also appears due to the Ag-Cl vibration. Since PYO is causing the aggregation of the nanoparticles, no additional salts were added to the colloid-analyte mixture. Thus, the bands in the low wavenumber range in the recorded spectra cannot be assigned to this vibration.

Based on the molecular structure of PYO (see Scheme 2), it is expected that oxygen and nitrogen atoms carry partial negative charges, thus, most probably an interaction with the silver surface is achieved via these heteroatoms. As a result of the interaction of PYO with Ag nanoparticles, Raman bands in the low wavenumber region, at 221 and 239 cm^−1^, can be observed and assigned to the Ag-N and Ag-O stretching vibrations [29]. The increase of their intensity (see Figure S5) can be attributed to the increment of the number of adsorbed PYO molecules on the Ag surface. The change of the ratio of these two bands with increasing PYO concentration might be caused by a change in the orientation of the target molecule on the surface of Ag nanoparticles. Namely, at low concentrations, the molecule might interact with the metallic surface via the free electron pairs of both N and O, while at high concentrations, due to the steric hindering, PYO might adopt an upright orientation and interact with the metallic surface only via the O atom.

The integrated peak area of the two convoluted bands in the low wavenumber range is presented by red scatter plot in Figure 3. Comparing it with the integrated peak area of the 676 cm^−1^ band, a similar sigmoidal behavior with saturation after 55 μM was observed. However, the underlying mechanism behind the increment of the signal is different for the two Raman bands. The Raman mode at 240 cm^−1^ band is due to the PYO molecules in the first layer on the surface of the silver nanoparticles. Meanwhile, the intensity of the 676 cm^−1^ is increasing also because of the molecules present in the upper layers but still found in the ‘hot-spot’ between the nanoparticles.

**Figure 4:**
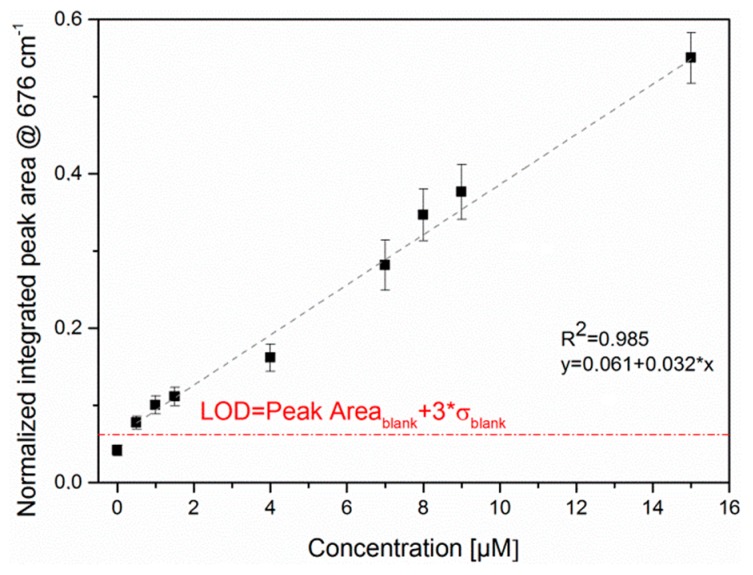
The peak area ratio of the 676 cm^−1^ and 240 cm^−1^ Raman modes as a function of PYO concentration in aqueous solution in the range between 0.5 and 15 μM with linear fitting. The red line indicates the calculated LOD.

In the following, the Raman band centered at 240 cm^−1^ was used as an internal standard [30]. The normalized integrated peak area of the Raman marker band of the metabolite is plotted in Figure 4. After normalization, a linear response over the 0.5–15 μM concentration range is achieved. According to the IUPAC definition, the limit of detection is equal with the signal of the blank plus three times its standard deviation. The calculated limit of detection (LOD) is below the lowest measured concentration (0.5 μM) (the threshold is illustrated in Figure 4). Moreover, the normalization of the signal significantly improved the “chip-to-chip” reproducibility. As illustrated in the supporting information, the integrated peak area of the Raman mode at 676 cm^−1^ for two different microfluidic chips is difficult to compare (Figure S6); however, after normalization, the concentration curves gain the same profile (Figure S7). Therefore, the Ag-adsorbed atom mode can be considered to be an internal standard and it increases the comparability of different data sets.

### 3.3. SERS Detection of PYO in Saliva

**Figure 5:**
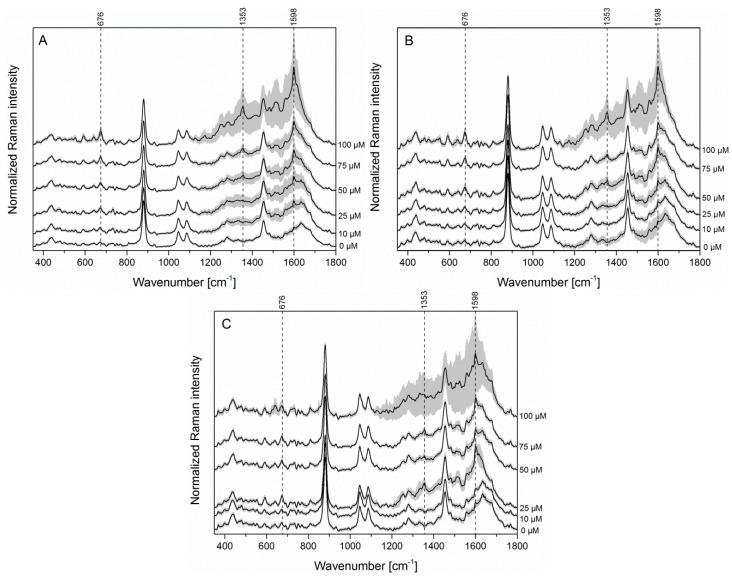
Mean SERS spectra and their double standard deviation of the different concentrations of PYO in the saliva sample from volunteer number one (**A**), two (**B**) and three (**C**).

In order to move toward clinical applications, the feasibility of SERS for PYO detection in saliva was investigated. The spectroscopic analysis of clinical samples is often challenging due to the complex and heterogeneous nature of biological fluids. In the case of SERS, different components can interact with the metallic structures and the detection of the Raman modes of the molecules of interest present in low concentrations can be inhibited. In the current study, saliva was chosen as the test sample matrix. This biological fluid will be present in the respiratory tract samples. Despite the fact that saliva is composed mainly of water, it has a multitude of dissolved components, for example minerals, electrolytes, hormones, mucins and enzymes. It also contains expectorated bronchial and nasal secretions, serum and blood derivatives from oral wounds, bacteria and bacterial products, viruses, fungi, desquamated epithelial cells and food debris [31]. Since the composition of saliva varies among individuals, for the proof of concept, saliva samples from three healthy volunteers were used for this study. Six different solutions with different PYO salivary concentrations (0 M, 10 μM, 25 μM, 50 μM, 75 μM, 100 μM) were prepared for each sample by mixing 9 parts of human saliva with 1 part of ethanol solution containing the target molecule. To remove oral epithelial cells and food debris, the samples were filtrated prior to the measurement. The recorded mean SERS spectra of the samples originating from the three volunteers at different PYO concentrations are presented in Figure 5. Since the ethanol concentration in all samples was kept constant, for a better comparison, all spectra were normalized to the ethanol Raman mode at 880 cm^−1^.

The SERS signal of the pure saliva (Figure S8) is challenging to interpret because of the convolution of the Raman bands in the fingerprint region. However, the Raman modes around 1240, 1472, 1610 and 1653 cm^−1^ can be distinguished and tentatively assigned to proteins, amino acids, lipids and DNA and/or RNA bases [32,33]. Because PYO stock solutions were prepared in ethanol, as negative control for the current study a mixture of saliva with ethanol was considered. The latter molecule gives rise to the intensive Raman bands at 880, 1047, 1087 and 1456 cm^−1^ observed in Figure 5 for the 0 μM samples. By spiking PYO to the complex matrix, a new band appears at 1353 cm^−1^, while the bands at 676 cm^−1^ and 1598 cm^−1^ gradually increase in intensity with the increment of PYO concentration. These three Raman modes are characteristic for the targeted molecule. In the case of the first and second volunteer, spectral changes can be seen already at the lowest investigated PYO concentration (10 μM) (Figure 5A,B and Figure S9), while for the third volunteer changes can be noticed by eye at a concentration of 25 μM (Figure 5C and Figure S9). In the case of the highest concentration, 100 μM, the increased standard deviation of the recorded spectra can be explained by the poisoning of the metallic surface by the high number of molecules present in the matrix. In comparison with the LOD previously obtained for PYO in water (below 0.5 μM), the complexity of the matrix affects the sensitivity of the method and the metabolite could be detected down to a 10 μM concentration. For these samples, the LOD was not calculated based on the IUPAC definition, but instead the lowest concentration causing observable changes in the SERS spectrum of the sample was considered. Nevertheless, by using easy to prepare silver nanoparticles, we showed that the detection of PYO in the clinical range can be achieved and the saliva collected from the three volunteers does not significantly affect the detection of the target molecule.

## 4. Conclusions

*P. aeruginosa* is an opportunistic human pathogen responsible for acute and chronic infections, particularly in individuals with compromised immune systems and host defenses. PYO is a virulence factor uniquely produced by this pathogen. Thus, its fast and selective detection can reveal the presence of *P. aeruginosa* in the organism, which can help with fast and successful antibiotic treatment.

In this study, the detection of pyocyanin (PYO) was performed through surface-enhanced Raman spectroscopy (SERS) combined with a microfluidic platform. It was shown that the aggregation of the silver nanoparticles is induced by the presence of the analyte and no additional salts are necessary to achieve enhancement. PYO was successfully detected in aqueous solution in the clinically relevant range (7–130 μM) with the linearity in the 0.5 μM–15 μM region and a limit of detection <0.5 μM. This demonstrated that the described LoC-SERS approach is versatile and allows the detection of bacteria metabolites. In the second part of the paper, as proof of concept, saliva collected from three volunteers was considered as a matrix. As none of the individuals were under treatment for pneumonia, PYO was artificially added to the saliva and it was successfully detected in this chemical environment down to the 10 μM concentration for two of the investigated samples, while in the case of the third sample spectral changes were observed at 25 μM. The results establish the potential for the detection of PYO directly from complex matrixes. For further studies, other respiratory specimens will be also considered. Additionally, the measurement principle used can be significantly improved by replacing the bench-top Raman setup with a miniaturized or even portable system, by integrating the sample clean-up step in the platform and by opting for a pressure driven pumping system.

## Supplementary Materials

The following are available online at http://www.mdpi.com/1424-8220/17/8/1704/s1.
