# Supplementary material for: Detection of Pseudomonas aeruginosa Metabolite Pyocyanin in Water and Saliva by Employing the SERS Technique
Source: Sensors (Basel). 2017 Jul 25;17(8):1704. doi: 10.3390/s17081704 (PMC5580190; doi:10.3390/s17081704)
Supplement: Supplementary file 1 [file sensors-17-01704-s001.pdf]

Supplementary material

Detection of *Pseudomonas aeruginosa* metabolite pyocyanin in water and artificial sputum by employing the LOC-SERS technique

Olga Žukovskaja [1,2], Izabella Jolan Jahn [3], Karina Weber [1,2,3], Dana Cialla-May [1,2,3,] and Jürgen Popp [1,2,3]*

1 Friedrich Schiller University Jena, Institute of Physical Chemistry and Abbe Center of Photonics, Helmholtzweg 4, 07745 Jena, Germany;
2 Research Campus Infectognostic, Philosophenweg 7, 07743 Jena, Germany;
3 Leibniz Institute of Photonic Technology Jena, Albert-Einstein-Str. 9, 07745 Jena, Germany;
* Correspondence: dana.cialla-may@leibniz-ipht.de; Tel.: +49-3641-206-309

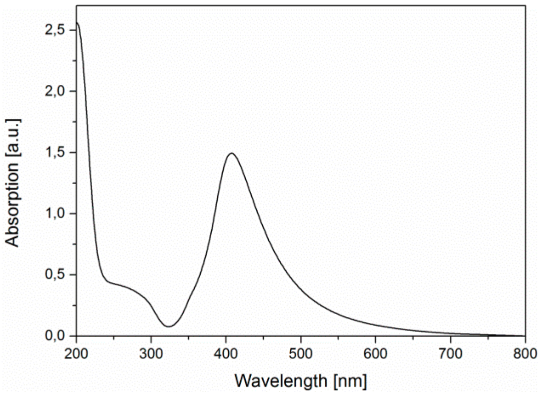

Figure S1. UV-Vis absorption spectra of the Ag nanoparticles.

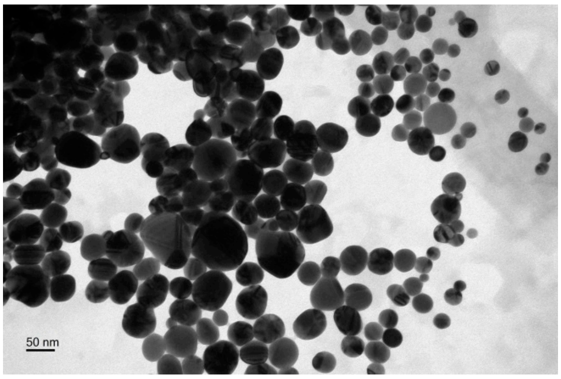

Figure S2. TEM image of Ag nanoparticles.

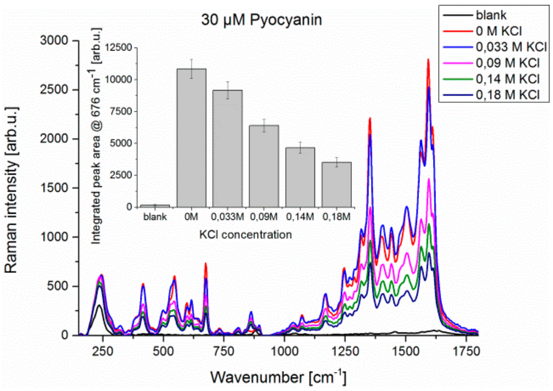

Figure S3. Mean SERS spectra of 30 µM PYO with different concentrations of KCl measured in the microfluidic platform. In the inset the integrated peak area of the PYO Raman mode at 676 cm^{-1} and its double standard deviation for different KCl concentrations is illustrated.

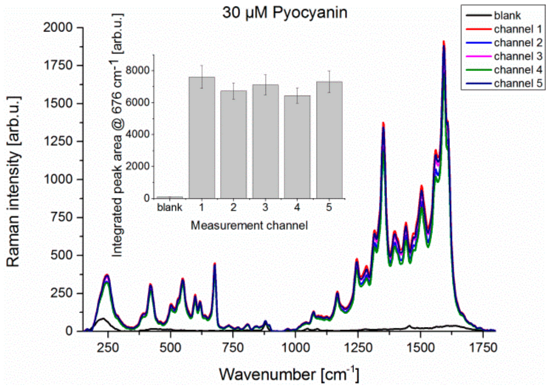

Figure S4. Mean SERS spectra of 30 µM PYO measured in different channels of the microfluidic chip. In the inset the integrated peak area of the PYO Raman mode at 676 cm^{-1} and its double standard deviation is illustrated.

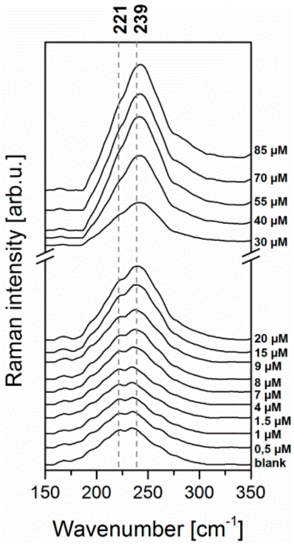

Figure S5. Mean SERS spectra of PYO with different concentrations in the region of Ag-O and Ag-N complexes vibrations.

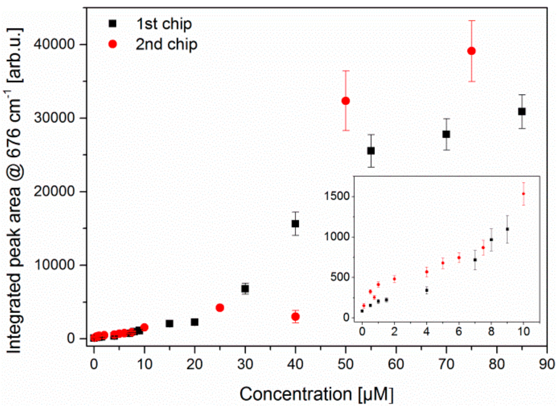

Figure S6. The peak area of the 676 cm^{-1} Raman mode as a function of PYO concentration for 2 different chips. In the inset zoomed area for concentrations till 10 µM.

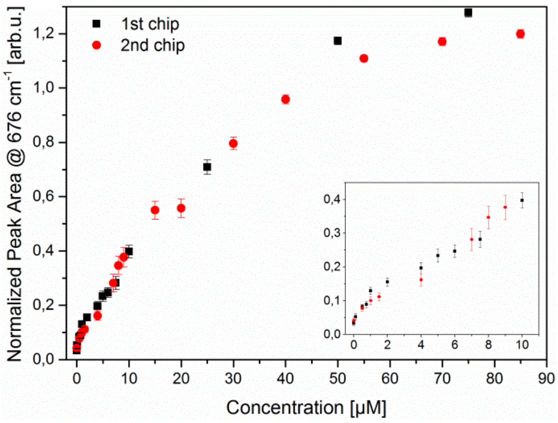

Figure S7. The peak area ratio of the 676 cm^{-1} and 240 cm^{-1} Raman modes as a function of PYO concentration. In the inset zoomed area for concentrations till 10 µM.

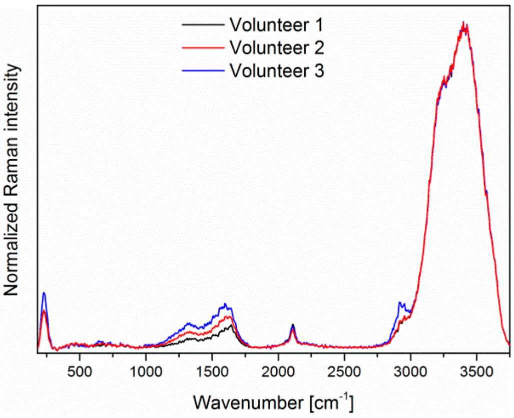

Figure S8. Mean SERS spectra of pure saliva of different volunteers

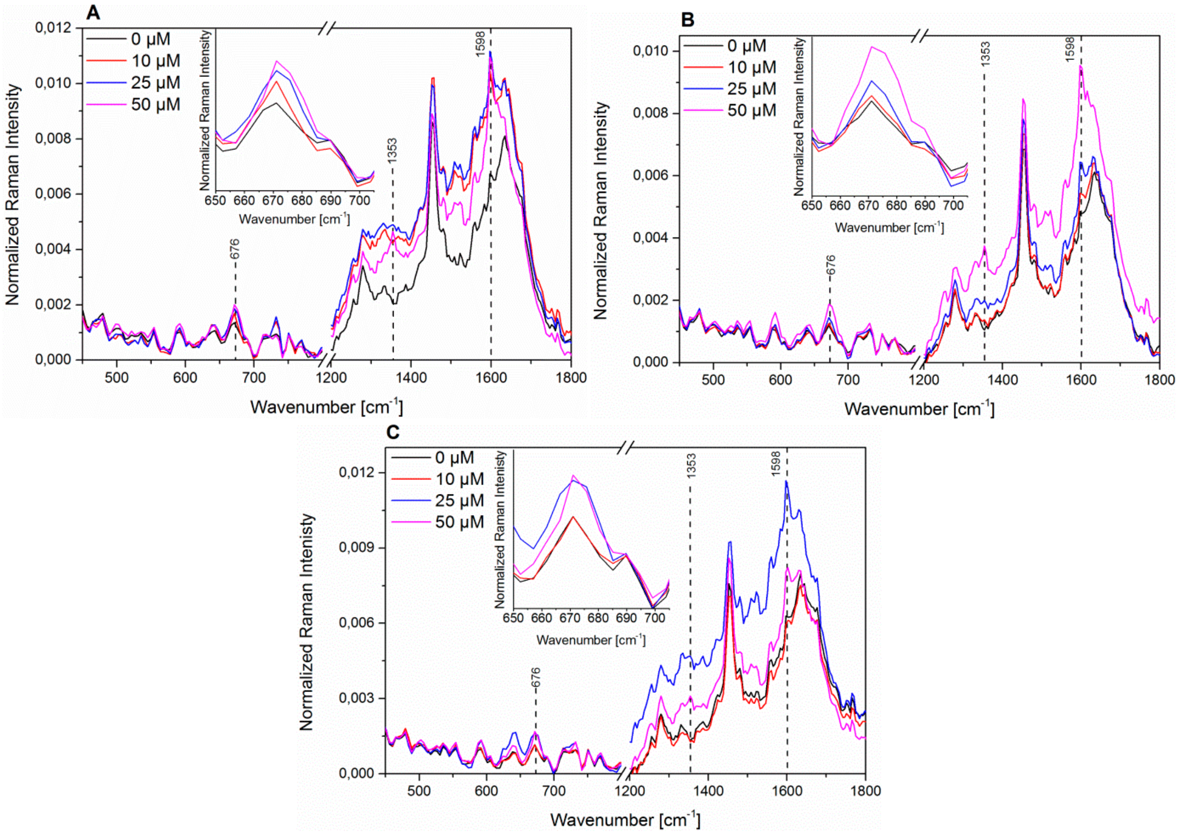

Figure S9. Mean SERS spectra in the fingerprint region of the three lowest concentrations of PYO in the saliva sample from volunteer number one (A), two (B) and three (C). In the inset the Raman band at 676 cm^{-1} is plotted.
